# Detection of dengue virus infection in children presenting with fever in Hawassa, southern Ethiopia

**DOI:** 10.1038/s41598-023-35143-2

**Published:** 2023-05-17

**Authors:** Techalew Shimelis, Andargachew Mulu, Mesfin Mengesha, Aynalem Alemu, Adane Mihret, Birkneh Tilahun Tadesse, Adam W. Bartlett, Fitsum W/Gebriel Belay, Gill Schierhout, Sabine Dittrich, John A. Crump, Susana Vaz Nery, John M. Kaldor

**Affiliations:** 1 College of Medicine and Health Sciences, Hawassa University, Hawassa, Ethiopia; 2 Armauer Hansen Research Institute, Addis Ababa, Ethiopia; 3 Kirby Institute, University of New South Wales, Sydney, Australia; 4 The George Institute for Global Health, University of New South Wales, Sydney, Australia; 5 Foundation for Innovative New Diagnostics, Geneva, Switzerland; 6 Nuffield Department of Medicine, University of Oxford, Oxford, UK; 7 Deggendorf Institut of Technology, European Campus Rottal-Inn, Deggendorf, Germany; 8 Centre for International Health, University of Otago, Dunedin, New Zealand

**Keywords:** Microbiology, Diseases, Signs and symptoms

## Abstract

Dengue fever is a mosquito-borne viral infection, with rising incidence globally. Eastern Ethiopia has had dengue fever outbreaks in recent years. However, the extent to which the infection contributes to hospital presentation among children with fever in southern Ethiopia is unknown. We examined 407 stored plasma samples collected to investigate the aetiology of fever in children aged at least 2 months and under 13 years presenting to the outpatient of the largest tertiary hospital in southern Ethiopia. We analyzed samples for dengue virus non-structural 1 antigen using enzyme-linked immunosorbent assay. The median (interquartile range) age of the 407 children examined was 20 (10–48) months, and 166 (40.8%) of the children were females. Of 407 samples analyzed, 9 (2.2%) were positive for dengue virus non-structural 1 antigen, of whom 2 were initially treated with antimalarial drugs despite having negative malaria microscopy, and 1 of the 8 patients had a persistent fever at the seventh day of follow-up time. The presence of active dengue virus infection in the study area highlights the need for studies at the community level as well as the integration of dengue diagnostics into fever-management strategies. Further research to characterize circulating strains is warranted.

## Introduction

The emergence and re-emergence of fever-causing, mosquito-borne viruses belonging to the genus *Flavivirus* such as dengue and yellow fever virus are global public health concerns^1^. An estimated 100–400 million dengue virus infections occur each year, with an increase over the past 20 years, from 505,430 illnesses and 960 deaths in 2000 to 5.2 million illnesses and 4032 deaths in 2019, with younger age groups most affected^2^. Asia accounts for about 70% of estimated global dengue infections, while Africa and the Americas represent 16% and 14%, respectively^3^. In Ethiopia, dengue outbreaks have been reported in the eastern part of the country since 2013^4,5^, but there is a lack of information on whether the infection is present in other administrative regions. In small-scale studies, seropositivity for anti-dengue IgM and IgG was reported in febrile patients to be 8.1% and 25.1%, respectively, in Arba Minch City, southern Ethiopia^6^, and 19% and 21%, respectively, in Northwest Ethiopia^7^.

Dengue is often diagnosed before day 5 of illness onset by viral RNA identification using reverse transcriptase-polymerase chain reaction (RT-PCR) and viral non-structural 1 (NS1) antigen detection via enzyme-linked immunosorbent assay (ELISA), but the former technique is more sensitive^8,9^. The detection of anti-dengue IgM and IgG antibodies by immunoassays is also helpful in the diagnosis^10^. However, the lower sensitivity of serological tests due to decreased antibody titres at the acute phase, lower specificity due to cross-reactivity with related flaviviruses, and longer blood persistence limits their usefulness in clinical management^11,12^.

In settings where there is limited laboratory capacity, guidelines recommend antibacterial treatment based on clinical case definitions, but parasitological confirmation of malaria is required before administering antimalarial medications^13,14^. However, health workers may disregard recommendations and unnecessarily prescribe antimalarials and antibacterials^15–17^.

In our recent study of infectious aetiologies of fever in children presenting to the largest tertiary hospital in Hawassa, southern Ethiopia, there was no laboratory evidence for any of the pathogens that were assessed in 61% of participants^18^. Due to resource constraints, the dengue virus was not included in the original panel of tests that we used for this study. We have subsequently been able to undertake dengue testing, and therefore we can now report the results in our cohort of febrile children.

## Methods

### Study setting and samples

The study setting and overall design have been previously reported in detail^18^. In brief, the study was conducted at Hawassa University Comprehensive Specialized Hospital (HUCSH), which is the largest in southern Ethiopia. Hawassa City is situated on the shore of Lake Hawassa at 1,708 m above sea level, with an annual temperature range of 9–29 °C (a mean monthly temperature of 19.7 °C), and a mean annual rainfall of 961 mm^19,20^. A consecutive series of 433 children aged at least 2 months to less than 13 years presenting to the hospital were prospectively enrolled from May 2018 through February 2019 to assess aetiologies of fever. Fever was defined as a temperature of 37.5 °C or a history of fever within the preceding 48 h, lasting no longer than 7 days. Critically ill patients for whom blood or urine cultures were not performed as part of their care at admission were excluded due to ethical reasons. In July 2022, stored plasma samples from 407 children were examined for the current study to make a retrospective diagnosis of dengue virus infection. Due to inadequate blood volume, plasma samples from the remaining 26 enrolled children were not stored.

### Diagnosis, clinical management, and outcome

As described in detail elsewhere^18,21^, hospital staff gathered clinical information from physical examinations and history-taking. Laboratory investigations including complete blood count (CBC), malaria microscopy, human immunodeficiency virus (HIV) testing, urinalysis and urine culture, and blood culture were performed for each participant. Other tests were performed based on relevant case presentations^18^. Clinicians managed participants on the day of enrolment in accordance with their presentation and results of malaria microscopy, CBC, and urinalysis. Within a week after enrolment, additional diagnoses and management were made based on urine or blood culture findings. Data on fever status were gathered on day 7. Records for each child were retrospectively reviewed by senior paediatricians, who determined whether antibacterial or antimalarial treatments had been indicated for the child at the time of presentation, based on Ethiopian national guidelines^22^. Case definitions for clinical and laboratory indicators are presented as supplementary information (Table S1).

### Serological analysis for the retrospective diagnosis of dengue virus infection

Plasma specimens that had been stored at − 70 °C for about 3 years were tested for the specific dengue non-structural protein 1 (NS1) at Armauer Hansen Research Institute in Addis Ababa, using the commercially available Dengue Virus NS1 ELISA (EUROIMMUN Medizinische Labordiagnostika AG, Lübeck, Germany). Briefly, the plasma sample was diluted 1:2 in sample buffer and added to microplate strip wells precoated with monoclonal anti-dengue virus NS1 antibodies against all the dengue serotypes and incubated for 1 h. After a washing step, peroxidase labelled anti-dengue virus NS1 antibody was added to the wells and incubated for 1 h followed by a washing step. Then, after the addition of the chromogenic substrate and incubation for 15 min, the stop solution was added. Shortly, a photometric measurement was made at 450 nm/620 nm and a semi-quantitative and quantitative determination of NS1 antigen was performed based on the absorbance of the samples and calibrators. In the semi-quantitative analysis, a result is considered positive when the ratio of extinction reading of a sample to a calibrator (standard) is ≥ 1.1. For the quantitative analysis, the concentration of NS1 in a sample in Relative Units (RU)/ml was determined from a standard curve plotted using extinction readings measured for the 3 calibration sera (standards) against the corresponding relative units; hence, a sample with ≥ 11 RU/ml was considered as positive. The test is claimed to have a 99% sensitivity and specificity, and is carried out in accordance with the manufacturer's instructions. An acute dengue virus infection was defined as the presence of NS1 antigen in the blood (NS1 antigenaemia).

### Data analysis

Statistical Package for Social Sciences version 20 (IBM Corp., New York, USA) was used for data entry and analysis. Z-scores for anthropometry were calculated using the WHO AnthroPlus software^23^. The detection of NS1 antigen in relation to demographic and clinical characteristics was expressed as frequencies with percentages. The crude odds ratio (cOR) for the evaluation of factors related to the prevalence of dengue virus infection was calculated using binary logistic regression analyses. An association was judged as statistically significant if the corresponding p-value was less than 0.05.

### Ethics approval and consent to participate

The University of New South Wales (Ref. No: HC180078) and the Hawassa University College of Medicine and Health Sciences (Ref. No: IRB/176/10) ethics committees approved the study. Children's caregivers were given adequate information about the study and written informed consent was obtained from all caregivers, in addition to assent from children aged at least 12 years. Code numbers were used in place of identifiers to ensure the confidentiality of collected information. All methods were performed in accordance with the relevant guidelines and regulations.

## Results

In July 2022, plasma samples collected previously from 407 (93.9%) of the 433 enrolled participants were tested for dengue NS1 antigen. The median (IQR) age of the 407 children tested was 20 (10–48) months, ranging from 2 months to 12 years, 166 (40.8%) of the children were females, and 97 (23.7%) were underweight (weight-for-age z-score, − 3 to < − 2). The 407 participants had a median (IQR) axillary temperature of 38 °C (37.6–38.5 °C), fever duration of 3 (1–4) days at enrolment, total WBC count of 10.5 (7.6–14.0) × 10^3^ cells/mm^3^, and haematocrit value of 39.1% (35.5–43%).

Dengue NS1 antigen was detected in 9 (2.2%) of 407 study participants, including 5 (3.0%) of 166 females, 1 (0.8%) of 122 infants (2–11 months), 6 (2.5%) of the 238 participants from Hawassa, 1 (1.7%) of the 58 from outside Hawassa, and 2 (1.8%) of the 111 from the neighbouring region of Oromia. Two (2.2%) of 93 underweight children, 0 (0%) of 85 stunted children, and 1 (0.9%) of 111 wasted children were found positive for dengue (Table 1).

**Table 1:** Distribution of dengue virus infection in febrile children attending HUCSH, 2018–2019.

Characteristics	Dengue virus infection			
	n (%) blood tested (N = 407)	n (%)^†^ positive	n (%)^†^ negative	cOR (95% CI)
Residence Adm.Region				
Hawassa	238 (58.5)	6 (2.5)	232 (97.5)	1.41 (0.28–7.10)
Outside Hawassa	58 (14.3)	1 (1.7)	57 (98.3)	0.96 (0.09–10.8)
Oromia	111 (27.3)	2 (1.8)	109 (98.2)	1
Gender				
Female	166 (40.8)	5 (3.0)	161 (97.0)	1.84 (0.49–6.96)
Male	241 (59.2)	4 (1.7)	237 (98.3)	1
Age				
2–11 months	122 (30.0)	1 (0.8)	121 (99.2)	0.29 (0.03–3.25)
12–35 months	140 (34.4)	5 (3.6)	135 (96.4)	1.30 (0.25–6.85)
36–59 months	73 (17.9)	1 (1.4)	72 (98.6)	0.49 (0.04–5.48)
≥ 5 years	72 (17.7)	2 (2.8)	70 (97.2)	1
Weight-for-age z-score*				
Underweight (− 3 to < − 2)	93 (23.7)^a^	2 (2.2)	91 (97.8)	0.92 (0.19–4.51)
Normal (≥ − 2)	300 (76.3)^a^	7 (2.3)	293 (97.7)	1
Height-for-age z-score				
Stunting (− 3 to < − 2)	85 (20.9)^b^	0	85 (100.0)	
Normal (≥ − 2)	321 (79.3)^b^	9 (2.8)	312 (97.2)	–
BMI-for-age z-score				
Wasting (− 3 to < − 2)	111 (27.3)^b^	1 (0.9)	110 (99.1)	0.33 (0.04–2.64)
Normal (≥ − 2)	295 (72.7)^b^	8 (2.7)	237 (97.3)	1

*cOR* crude odds ratio, *CI* confidence interval, *BMI* body-mass-index.

*Weight-for-age was calculated only for children up to 10 years of age, ^a^(N = 393), ^b^(N = 406).

^†^Percentages within categories of the characteristics (raw total).

Dengue was detected in 3 (3.9%) of 76 children with a fever of 5–7 days at presentation, 5 (2.3%) of 218 with a cough, 2 (2.6%) of 76 with diarrhoea, 3 (2.0%) of 148 with vomiting, and 2 (3.4%) of 58 with an axillary temperature of at least 39 °C. Moreover, 2 (4.2%) of 48 children with anaemia and 0 (0%) of 38 children with leukopenia were positive for dengue (Table 2). The presence of NS1 antigen was not significantly associated with any demographic or clinical characteristic of the participants (Tables 1 and 2).

**Table 2:** Distribution of dengue virus infection by clinical characteristics and laboratory findings of children attending HUCSH, 2018–19.

Characteristics	Dengue virus infection			
	n (%) blood tested (N = 407)	n (%)^†^ positive	n (%)^†^ negative	cOR (95% CI)
Clinical history				
Duration of fever				
1–4 days	331 (81.3)	6 (1.8)	325 (98.2)	1
5–7 days	76 (18.7)	3 (3.9)	73 (96.1)	2.23 (0.54–9.12)
Symptoms				
Cough				
Yes	218 (53.6)	5 (2.3)	213 (97.7)	1.09 (0.29–4.10)
No	189 (46.4)	4 (2.1)	185 (97.9)	1
Diarrhoea				
Yes	76 (18.7)	2 (2.6)	74 (97.4)	1.25 (0.26–6.14)
No	331 (81.3)	7 (2.1)	324 (97.9)	1
Vomiting				
Yes	148 (36.4)	3 (2.0)	145 (98.0)	0.85 (0.22–3.54)
No	259 (63.6)	6 (2.3)	253 (97.7)	1
Vital signs				
Axillary temperature				
< 37.5 °C	44 (10.8)	1 (2.3)	43 (97.7)	1
37.5–38.9 °C	305 (75.2)	6 (2.0)	229 (98.0)	0.86 (0.10–7.34)
≥ 39 °C	58 (10.8)	2 (3.4)	56 (96.6)	1.54 (0.14–17.5)
Tachypnea				
Yes	233 (57.2)	5 (2.1)	228 (97.9)	0.93 (0.25–3.52)
No	174 (42.8)	4 (2.3)	170 (97.7)	1
Tachycardia				
Yes	163 (40.0)	4 (2.5)	159 (97.5)	1.20 (0.32–4.55)
No	244 (60.0)	5 (2.0)	239 (98.0)	1
Lab analysis				
WBC count				
Leukopenia	38 (9.4)^c^	0	38 (100.0)	–
Leucocytosis	66 (16.3)^c^	1 (1.5)	65 (98.5)	0.56 (0.07–4.57)
Normal count	300 (74.3)^c^	8 (2.7)	292 (97.3)	1
Anaemia				
Yes	48 (11.8)^c^	2 (4.2)	46 (95.8)	2.17 (0.44–10.8)
No	356 (88.1)^c^	7 (2.0)	349 (98.0)	1

*cOR* crude odds ratio, *CI* confidence interval, *WBC* white blood cell.

^†^Percentages within categories of the characteristics (raw total), ^c^(N = 404).

As shown in Table 3, of 9 children who tested positive for NS1, 4 were diagnosed with pneumonia, 1 with tonsillopharyngitis, and 2 with acute diarrhoea at the time of initial management on enrolment day. When the culture results were released following initial management, 2 of the 8 children with urine cultures and 1 of the 9 children with blood cultures had urinary tract infections (*Escherichia coli*, *Klebsiella pneumoniae*) and bacteraemia (*Staphylococcus aureus*), respectively.

**Table 3:** Dengue virus infection by types of previous diagnoses of febrile children attending HUCSH, 2018–2019.

Previous diagnosis	Dengue virus infection		
	n (%)^‡^ positive (N = 9)	n (%)^‡^ negative (N = 398)	n (%) Total (N = 407)
Pneumonia			
Yes	4 (–)	166 (41.7)	170 (41.8)
No	5 (–)	232 (58.3)	237 (58.2)
Tonsilopharyngitis			
Yes	1 (–)	41 (10.3)	42 (10.3)
No	8 (–)	357 (89.7)	365 (89.7)
Acute diarrhoea			
Yes	2 (–)	74 (18.6)	76 (18.7)
No	7 (–)	324 (81.4)	331 (81.3)
Malaria			
Yes	0	14 (3.5)	14 (3.4)
No	9 (–)	384 (96.5)	393 (96.6)
Bacteraemia			
Yes	1 (–)	23 (5.9)^d^	24 (6.0)^e^
No	8 (–)	366 (94.1)^d^	374 (94.0)^e^
Urinary tract infections			
Yes	2 (–)	68 (18.3)^g^	70 (18.5)^h^
No	6 (–)	303 (81.7)^g^	309 (81.5)^h^
Undifferentiated fever			
Yes	0	58 (14.6)	58 (14.3)
No	9 (–)	340 (85.4)	349 (85.7)

^‡^Percentages within column total; ^d^(N = 389), ^e^(N = 398), ^g^(N = 371), ^h^(N = 379).

Although all 9 NS1-positive participants were negative for malaria, 2 of the 9 children had initially been given antimalarial medication. Based on the empiric guidelines, 6 of the 9 children were judged to have initial clinical indications for antibacterial drugs, but 7 of the 9 children received this treatment (Table 4).

**Table 4:** Dengue virus infection by initial clinical management and day 7 outcomes of febrile children attending HUCSH, 2018–2019.

Characteristics	Dengue virus infection		
	n (%)^‡^ positive (N = 9)	n (%)^‡^ negative (N = 398)	n (%) Total (N = 407)
Initial management at baseline			
Managed as inpatient	1 (–)	153 (39.1)^i^	154 (38.5)^k^
Managed as outpatient	8 (–)	238 (60.9)^i^	246 (61.5)^k^
Prescriptions and adherence to guidelines			
Had clinical indications for antibacterial therapy			
Yes	6 (–)	304 (77.7)^i^	310 (77.5)^k^
No	3 (–)	87 (22.3)^i^	90 (22.5)^k^
Prescribed antibacterials			
Yes	7 (–)	326 (83.4)^i^	333 (83.2)^k^
No	2 (–)	65 (16.6)^i^	67 (16.8)^k^
Over prescribed antibacterials			
Yes	1 (–)	30 (7.7)^i^	31 (7.8)^k^
No	8 (–)	361 (92.3)^i^	369 (92.2)^k^
Over prescribed antimalarials			
Yes	2 (–)	23 (5.9)^i^	25 (6.2)^k^
No	7 (–)	368 (94.1)^i^	375 (93.8)^k^
Day 7 follow-up			
Fever status			
Resolved	7 (–)	329 (90.1)^m^	336 (90.1)^n^
Persisted	1 (–)	36 (9.9)^m^	37 (9.9)^n^

^‡^Percentages within column total; ^i^(N = 391), ^k^(N = 400), ^m^(N = 365), ^n^(N = 373).

On day 7 of the follow-up, 8 of the 9 NS1-positive children were contacted. Of these, the caregiver of one child reported persisting fever, and 7 reported that fevers had subsided within 3 days following initial management (Table 4).

## Discussion

To our knowledge, this is the first report of acute dengue virus infection (NS1 antigenaemia) in southern Ethiopia. We found that 2.2% of febrile children presenting to a tertiary hospital in southern Ethiopia were positive for dengue NS1 antigen, an early marker of acute dengue virus infection. Compared to our finding, dengue IgM, a sign of recent infection, was seen in all ages of patients in southern (8.1%) and Northwest Ethiopia (19%)^6,7^. The discrepancy seen may be because previous studies tested for dengue-specific IgM, which is detectable for about 3 months after the onset of illness as opposed to the NS1 antigen, which is detectable for 1–2 weeks^9–11^. Moreover, the participants in the current study were children with localized or non-localized febrile illnesses who consecutively presented to a tertiary hospital, as opposed to earlier studies when all ages of patients without any clear clinical focus of infection or suspected of dengue infection were examined.

Studies in non-outbreak settings showed higher percentages of NS1 detection than we did, with NS1 antigen being found in 4.4% of all ages outpatients with undifferentiated fever in the Democratic Republic of the Congo^24^, and 6.1% of children under 15 years of age with dengue-related symptoms in Cameroon^25^. While a prevalence of NS1 antigenaemia as high as 35% is seen in all age febrile patients in Nigeria^26^, a prevalence as low as 2.2% in the same nation^27^ is reported that is compatible with our observation. An NS1 antigen prevalence of 24.1% is shown in a systematic review among dengue suspected cases in Southeast Asia^28^. Overall, the findings suggest that dengue, as an endemic and epidemic-prone arboviral infection, likely plays a varying role as a cause of illness in both place and time.

The ecological environment and climatological changes and patterns such as seasonality influence the breeding and abundance of *Aedes* mosquito vectors and contribute to the heterogeneity of the infection among African residents^29^. However, the limited studies that are available in Ethiopia make it difficult to comprehend the dynamics of dengue and urge further investigations into its epidemiology. The reported low proportion of dengue at the study hospital, which is located close to Lake Hawassa where mosquito-borne infections are expected to be common, may be explained by the hospital's regional referral function, which draws severe cases such as severe pneumonia, as shown in our initial report^21^. It is also possible that caregivers were more likely to seek care from lower-health facilities than the hospital when they suspected malaria and similar acute febrile illness, as observed in our recent report^30^. As a result, it would likely be more representative to expand the scope of such studies to include lower-level health facilities to capture mild acute febrile illnesses.

Early diagnosis of dengue is essential to reduce the risk of complications and prevent the spread of the virus^9^. Given the growing body of evidence showing dengue outbreaks in Ethiopia^4,5^, introducing rapid dengue testing aids to make appropriate fever management. Specifically, determining a viral aetiology of fever may minimize the unnecessary prescription of antibacterial and antimalarial drugs. Despite the small number of dengue cases in the current study, we were still able to observe antimalarial treatment being given to children who had tested negative for malaria but later found positive for dengue.

One of the limitations of this study was that dengue markers other than the NS1 antigen were not examined. The analysis of the samples for dengue RNA using more sensitive techniques such as RT-PCR might have recovered additional dengue cases and may have allowed for the identification of circulating dengue serotypes^9,12^. Anti-dengue IgM and IgG detection could have provided information on the level of recent and previous dengue infections circulating in the community^10^. The small number of dengue cases detected precluded conclusions about associated clinical characteristics. Dengue was not attempted to be categorized by severity. The generalizability of results to all febrile children may be constrained by selection bias arising from an institution-based study.

## Conclusion

A low prevalence of acute dengue virus infection was observed in febrile children presenting to a tertiary hospital. However, dengue was commonly misdiagnosed as other febrile illnesses and goes unnoticed, leading to an overuse of antimalarial and antibacterial medications. Our findings suggest that dengue needs to be adequately highlighted, including the importance to integrate dengue diagnostics into fever-management approaches. Further study is urgently required into the level of transmission at the community level and ecological factors associated with dengue fever to lessen the threat of outbreaks and characterise circulation strains.

## Supplementary Information

Supplementary Table S1.

## Data Availability

The datasets generated and/or analyzed during the current study are available from the corresponding author, upon reasonable request and with the Institutional Review Board of the Hawassa University College of Medicine and Health Sciences. Restrictions apply to the availability of these clinical data, which caregivers had consented for the collected information to be used for our research study only, and so are not publicly available.
