# Supplementary material for: Detection of dengue virus infection in children presenting with fever in Hawassa, southern Ethiopia
Source: Sci Rep. 2023 May 17;13:7997. doi: 10.1038/s41598-023-35143-2 (PMC10192357; doi:10.1038/s41598-023-35143-2)
Supplement: Supplementary file 1 — Supplementary Table S1. [file 41598_2023_35143_MOESM1_ESM.docx]

Table S1: Case definitions for clinical and laboratory indicators of febrile children attending HUCSH, 2018-19

| Infant | Children who are under 12 months old |
| --- | --- |
| ***Clinical signs*** |  |
| Tachycardia | A high pulse rate for age (age: 2-11m, >160; 12-47m, >130 beats/min; 48m-5y, >120 beats/min; 6-8y, >115 beats/min; 9-12y, >110 beats/min) |
| Tachypnea | A high respiratory rate for age (age: 2-11m, ≥50 breaths/min; 12-59m, ≥40 breaths/min; 5-12y, ≥30 breaths/min) |
| ***Clinical Diagnoses*** |  |
| Pneumonia | A history of cough and/or difficulty breathing, plus signs of (a) tachypnea OR (b) chest findings OR (c) auscultatory findings OR (d) radiologic findings |
| Tonsillopharyngitis | Presentation with (a) pharyngeal redness and enlarged tonsils or (b) neck lymph node and enlarged tonsils or (c) tonsillar exudate, which are suggestive of bacterial infection based on the national guidelines |
| Acute diarrhoea | Presentation with diarrhoea (stool frequency > 3 loose or liquid stools per day on at least one day in the week prior to enrolment) lasting less than 14 days |
| Undifferentiated fever | Cases with no identified source of infection for the fever on clinical and laboratory investigations conducted. |
| ***Laboratory diagnoses*** |  |
| Anaemia | A low haematocrit value for age (age: 2m, <28%; 3-6m, <29%; 7-24m, <33%; 25m-6y, <34%; 7-12y, <35%) |
| Leucocytosis | A high total white blood cell count for age (age: 2-6 months (m), >17500 cells/µl); 7-24m, >17000 cells/µl; 25-59m, >15500 cells/µl; 5-8 year (y), >14500 cells/µl; 9-12y, >13500 cells/µl) |
| Leukopenia | A low total white blood cell count for age (age: 2-24m, <6000 cells/µl; 25-59m, <5500 cells/µl; 5-8y, <5000 cells/µl; 9-12y, <4500 cells/µl) |
| Malaria | The presence of an asexual *Plasmodium* species on blood smear microscopy |
| Bacteraemia | A positive blood culture for pathogenic bacteria was defined as bacteraemia |
| Urinary tract infection | Urine culture showing significant bacteriuria (≥10^5^ and ≥10^4^ colony-forming-unit/ml of urine collected by clean catch and urethral catheterization, respectively) |
| Dengue virus infection | Positive dengue NS1 antigen. |
| ***Clinical outcomes*** |  |
| Resolved fever | Absence of fever for 2 consecutive days prior to day 7(±1) as reported by caregivers or measured temperature of 36.4ºC -37.5ºC. |
| Persisting fever | Fever episode within 2 days prior to day 7(±1) as reported by caregivers or measured temperature of ≥37.5ºC. |
